# Ultrasonic microbubbles promote mesenchymal stem cell homing to the fibrotic liver via upregulation of CXCR4 expression

**DOI:** 10.1186/s13008-023-00104-8

**Published:** 2024-02-24

**Authors:** Heming Xu, Yize Huang, Fasu Zhang, Wei Shi, Yan Cheng, Kai Yang, Pingping Tian, Fei Zhou, Yuan Wang, Xueqing Fang, Youliang Song, Bo Liu, Liwei Liu

**Affiliations:** 1 Department of Infectious Diseases, Tongling People’s Hospital, Tongling, 244000 Anhui China; 2 College of Medical Technology, Anhui Medical College, Hefei, Anhui China; 3 Department of Infectious Diseases, The 901th Hospital of PLA Joint Logistic Support Force, Hefei, 230031 Anhui China

**Keywords:** Mesenchymal stem cell, Ultrasound microbubble, Liver fibrosis, Homing, CXCR4, Preface

## Abstract

**Objective:**

To investigate the mechanism of ultrasound microbubbles (UTMB) promoting stem cells homing to fibrotic liver.

**Methods:**

Bone marrow derived mesenchymal stem cells (BMSCs) were divided into 5 groups with or without ultrasound microbubbles and continuously irradiated with ultrasound conditions of frequency 1 MHZ and output power 0.6 W/cm^2^ for different times, and then injected into a mouse model of liver fibrosis through the tail vein with or without ultrasound microbubbles, with sound intensity. The effect of ultrasound microbubbles on MSC expression of CXC chemokine receptor 4 (CXCR4) and homing fibrotic liver was evaluated by flow cytometry (FCM), western blot (WB) and immunohistochemistry (IHC) analysis.

**Results:**

The level of CXCR4 expression was significantly higher in the ultrasound microbubble group than in the non-intervention group (P < 0.05), and the number of MSC and the rate of CXCR4 receptor positivity in the ultrasound microbubble-treated liver tissues were significantly higher than in the non-intervention group (P < 0.01).

**Conclusion:**

Ultrasonic microbubbles can promote the expression of CXCR4 on the surface of MSCs, thus improving the homing rate of MSCs in fibrotic liver.

## Background

Cirrhosis is the terminal stage of various chronic liver diseases that responds poorly to medical conservative treatment [1]. Recent studies showed that mesenchymal stem cell transplantation is an effective therapy for liver fibrosis because of their low immunogenicity and multi-directional differentiation, and may prevent or slow the progression of liver fibrosis [2, 3]. The previous studies in our group showed that the transplantation of MSCs may improve liver function and coagulation of patients with decompensated cirrhosis [4]. However, present studies showed that many of the cells gathered in the lungs firstly, gradually increased in liver and spleen, and the liver has only few cells in liver after peripheral vein infusion of MSCs [5]. Thus, increasing the colonization rate of mesenchymal stem cells is of great significance in improving the therapeutic effect of patients with cirrhosis.

Successful MSCs therapy requires that the cells migrate from vascular endothelial to target tissues and colonize survival—a process called homing [6]. Previous research reports established that the stromal derived factor (SDF)-1/CXCR4 is the most important signaling pathway for mediating specific migration of MSCs to damaged tissues (Whether MSCs are derived from mouse or human bone marrow or fat or umbilical cord) [7–10]. Potter underwent bioinformatic analysis utilizing targetscan.org and mirdb.org showed SDF-1 related to stem cell migrate through miRNA regulation [11]. Tissue inflammation increases the expression of SDF-1, MSCs migrate to the injured tissue along the SDF-1 concentration gradient for repair [12]. The previous studies showed that intracellular CXCR4 expression levels are higher, but cell membrane receptor-positive cells are less than 1% [13], which has limited the effective implementation of MSCs-based strategies. Therefore, one potential hypothesis states that increasing the expression of MSCs membrane CXCR4 can chemise more MSCs into the liver for a better therapeutic effect.

It has been reported that the cavitational effect of ultrasound by disrupting microbubbles may cause mechanical stretching of the vessel wall and induce microvesicles to enter tissues through the intercellular space between endothelial cells [14]. Studies have shown that significant increases in VEGF, SDF-1, VCAM-1 and IL-1 were found in the local microenvironment induced by MSCs combined with ultrasound-targeted microbubble destruction (UTMD). Also, UTMB has been reported to increase the proportion of MSCs with surface CXCR4 in either in vitro or in vivo experiments [15].

However, there have been few reports on the mechanism of using ultrasound microbubbles to promote the homing of MSCs to treat cirrhosis. Based on the SDF-1/CXCR4 axis regulates the migration of MSCs, we hypothesized that the combination tail vein transplantation with ultrasound-mediated microbubble destruction could improve the limited MSC tropism for fibrotic liver. To test our hypothesis, we detected the expression of cell membrane CXCR4 after UTMB treatment. Also, we established a fibrosis model in mice and counted the number of homing MSCs labeled by green fluorescent protein (GFP) in the fibrosis liver of mice that were treated with UTMB combined with intravenous infusion of MSCs. The goal of this study was to identify the potential mechanism whereby UTMB improves the migration and homing of systemically implanted MSCs following fibrosis liver.

## Results

### The identification of microbubble (SonoVue®) and Mouse model of liver fibrosis

**Fig. 1:**
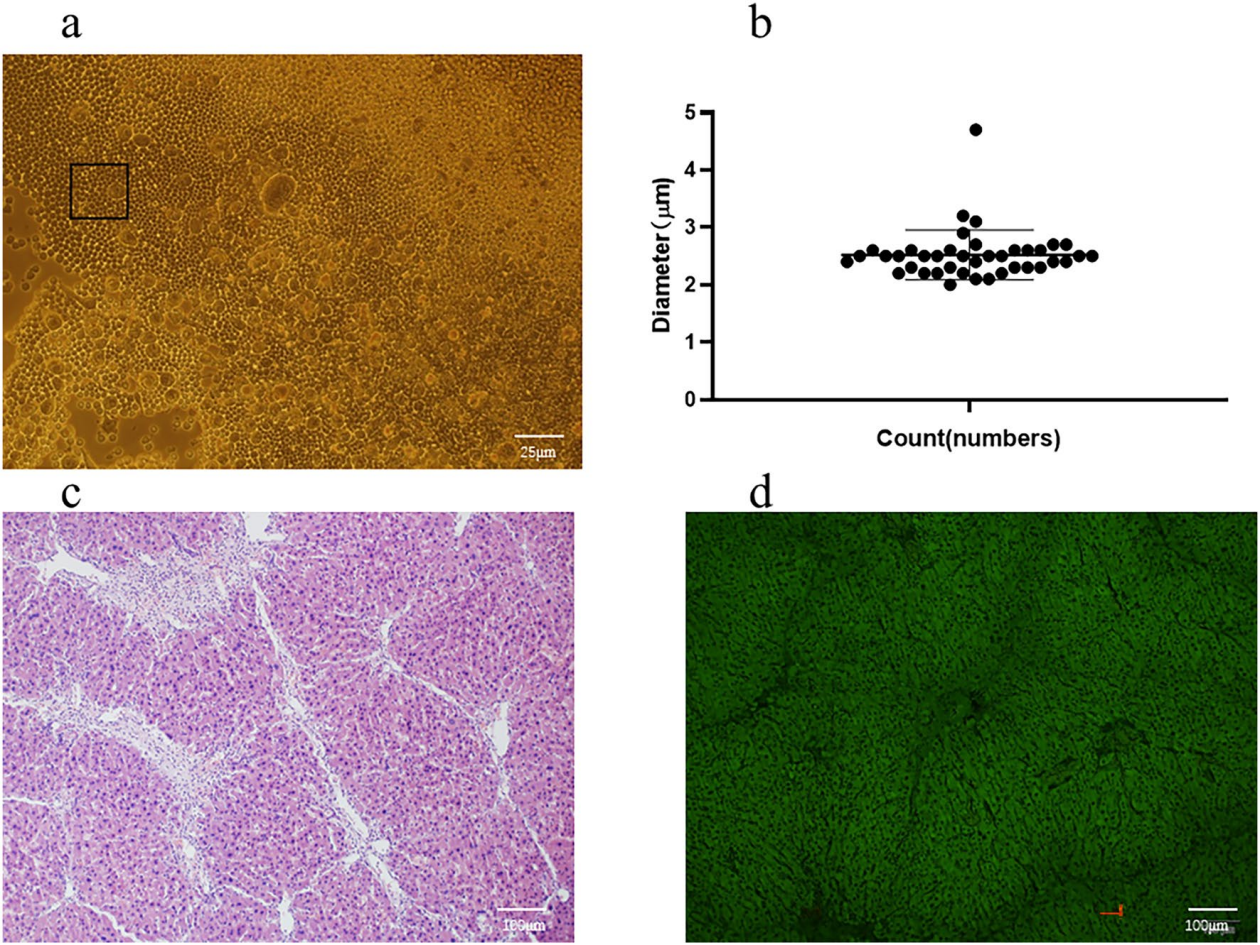
The identification of microbubble (SonoVue®) and Mouse model. **a** all microbubbles are less than 5 μm (n = 40, ×100, scale bar = 25 μm). **b** most microbubble size distribution Between in 2.10–4. 70 μm, the average diameter of microbubbles is about 2.52 ± 0.43 μm.** c** The mice livers with H&E staining had visible fibrosis, destruction of hepatic lobules, a large amount of hepatocyte apoptosis, and infiltration of inflammatory cells (n = 10, HE ×100, scale bar = 100 μm). **d** Fluorescence microscopy of HE staining of mouse liver fibrosis

SonoVue® is a lyophilized powder dosage form, each bottle contains SF6 (sulfur hexafluoride) gas 59 mg, lyophilized powder 25 mg. In the lyophilized powder added normal saline for injection, then shake vigorously, you can produce sulfur hexafluoride microbubbles, microbubble size distribution is relatively concentrated, all microbubbles are less than 5 μm (Fig. 1a), most microbubble size distribution between in 2.10–4.70 μm, the average diameter of microbubbles is about 2.52 ± 0.43 μm, (Fig. 1b) is a scatter plot of individual values for 40 independent microbubble diameters randomly circled from Fig. 1a. Liver fibrosis and destruction of hepatic lobules with visible massive hepatocyte apoptosis and inflammatory cell infiltration were found in H&E-stained mice livers 7 weeks after CCl4 administration, Independent pathologists scored liver lesions according to the semiquantitative metavir score, all enrolled experimental mice achieved F3 degree of fibrosis (Fig. 1c). Image of mouse liver fibrosis under HE-stained fluorescence microscope (Fig. 1d).

#### Culture and identification of mice BMSCs

**Fig. 2:**
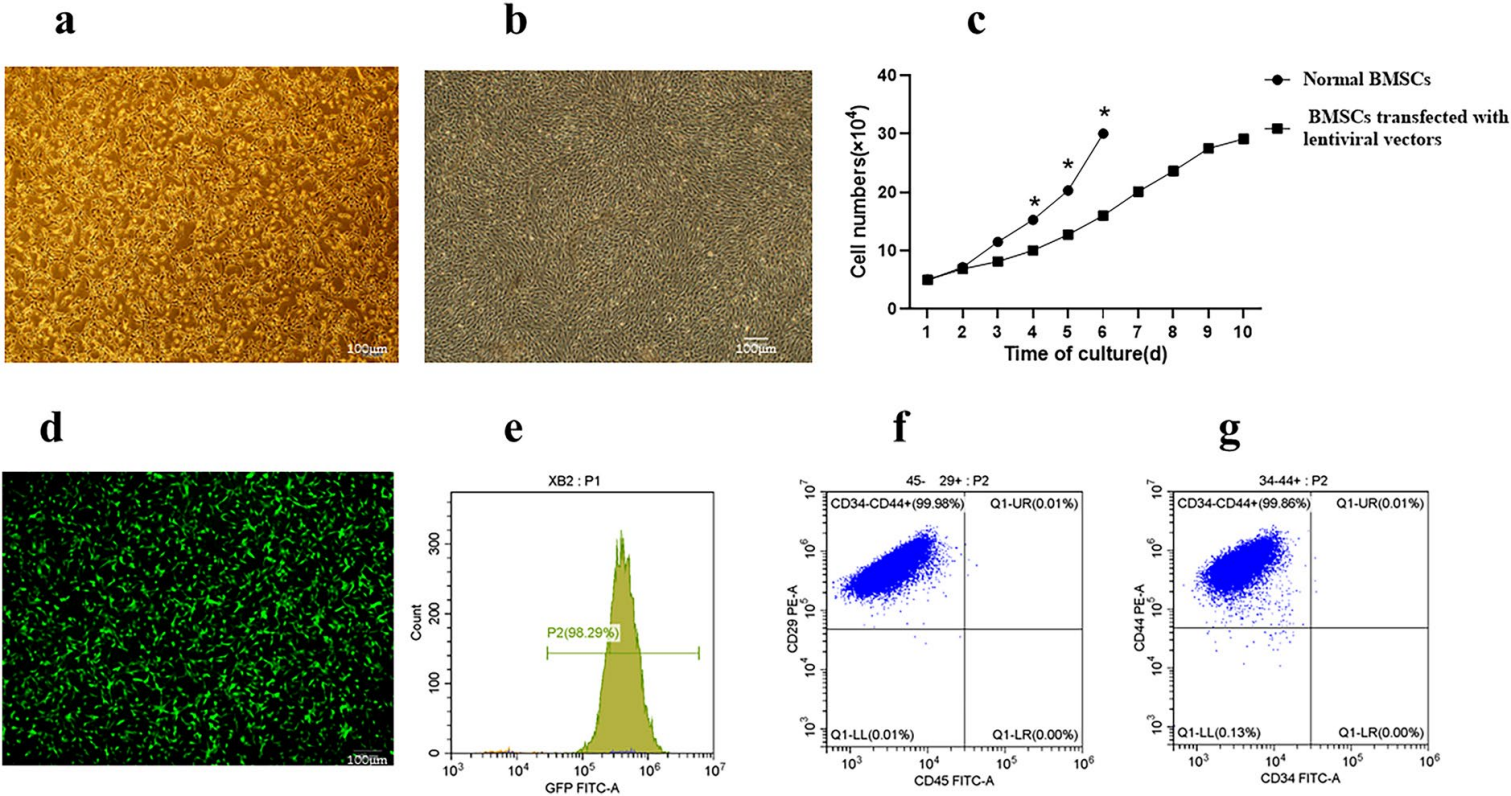
Characteristics of mice MSCs (n = 3). **a** BMSCs transfected with lentiviral vectors were cultured until day 6, The adherent cells reached approximately 80% confluence (×100, scale bar = 100 μm). **b** Normol BMSCs were cultured until day 6, The adherent cells approximately 100% confluence (×100, scale bar = 100 μm). **c** The normal BMSCs have better proliferative capacity(*, P < 0.05), Otherwise, The BMSCs transfected with lentiviral vectors can eventually multiply in sufficient numbers. **d** MSCs were labeled with GFP (×100, scale bar = 100 μm). **e** FCM revealed that the GFP expression level in BMSCs was approximately 97.21 ± 0.19%. **f** FCM analysis indicated that the positive rates of MSCs-specific antigens CD29 and CD44 were 97.2 and 97.8%. **g** The positive rates of CD34 and CD45 were 2.06% and 2.74%

The mice BMSCs at passages 3 were spindle-shaped, and then the mice BMSCs with lentivirus infected or uninfected were digested with 0.25% pancrepsin for 1–3 min to make a single-cell suspension, and then seed into a true diameter 90 mm dish at a density of 5 × 10^4^ pcs/ml, 3 dishes per group, total number of cells per day until the adherent cells reached approximately 100% confluence. BMSCs transfected with lentiviral vectors were cultured until day 6, the adherent cells reached approximately 80% confluence (Fig. 2a). The normal BMSCs adherent cells approximately 100% confluence (Fig. 2b). The normal BMSCs have better proliferative capacity, the BMSCs transfected with lentiviral vectors can eventually multiply in sufficient numbers (Fig. 2c). BMSCs transfected by lentiviral vectors carrying GFP exhibited bright green fluorescence in the cytoplasm and nucleus under a fluorescent microscope (Fig. 2d). Flow cytometry (FCM) revealed that the GFP expression level in BMSCs was approximately 98.21 ± 0.19% (Fig. 2e). Additionally, FCM analysis indicated that the positive rates of MSCs-specific antigens CD29 and CD44 was 99.9 and 99.8% (Fig. 2f); the specific antigens CD34 and CD45 of other cell lineage were not expressed in the MSCs, and the positive rate of these genes was 0.01% (Fig. 2g).

#### UTMB enhances the expression of CXCR4

**Fig. 3:**
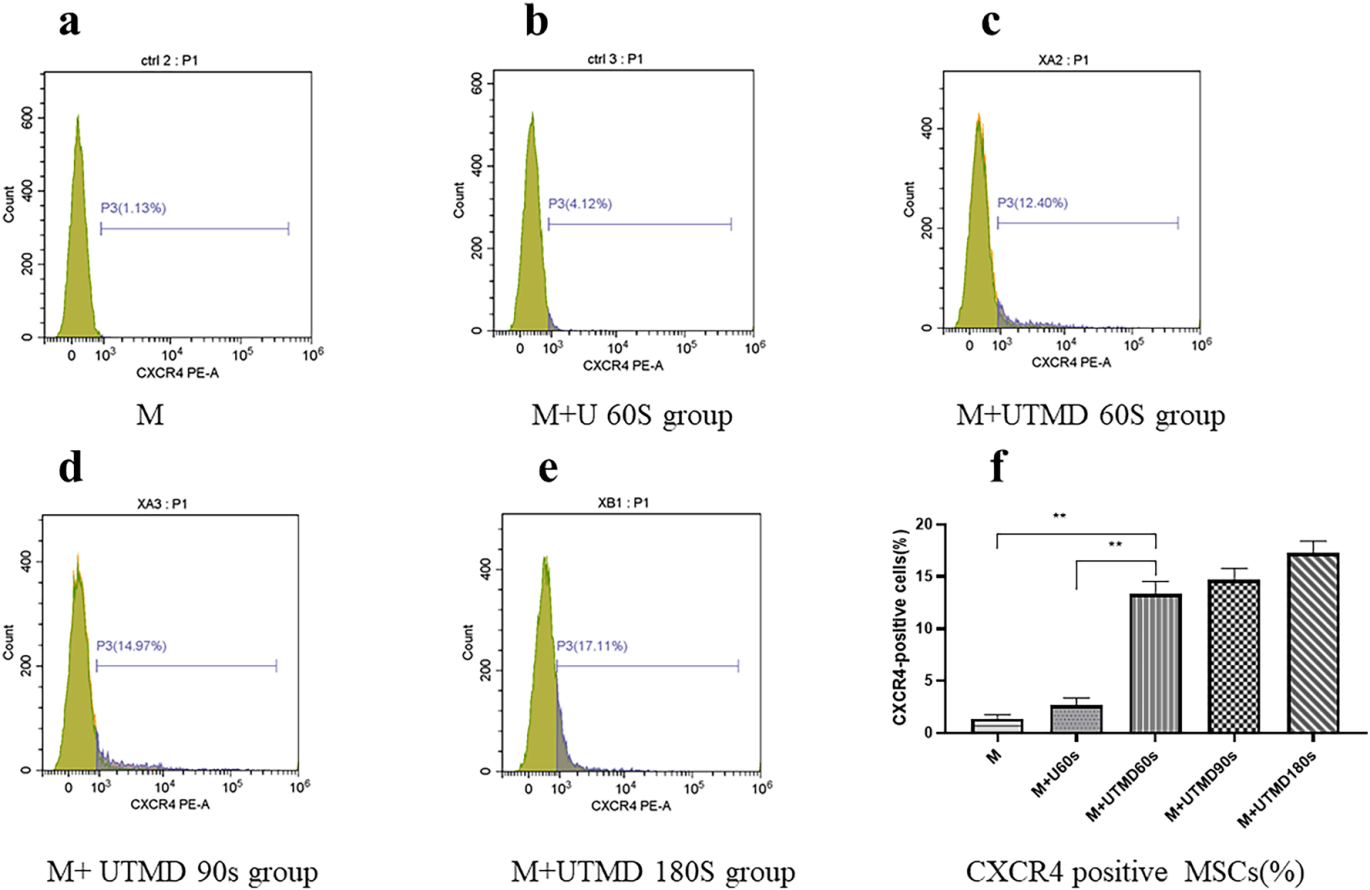
Cell surface expression of CXCR4 using FCM (n = 3). **a**–**e** Representative examples of the membrane expression levels of CXCR4 on MSCs in the different groups. The number of CXCR4-positive cells in the M + UTMB60s group (**c**) was significantly higher compared with the M group(a) and the M + U60s group (**b**). There was no significant difference in the number of CXCR4-positive cells between the M + UTMB60s group (**c**), the M + UTMB90s group (**d**) and the M + UTMB180s group (**e**). **f** Quantification of CXCR4 expression by FCM assay in the experimental MSCs. All values are expressed as the mean ± SD. **P < 0.01; *n* = 3

Flow cytometry detection showed that the percentage of cells expressing surface CXCR4 in the M + UTMB 180 s group (17.44 ± 1.16%) higher than the M + U 60 s group (2.67 ± 0.69%) and the M group (1.39 ± 0.37%) (Fig. 3a–e). As showed in Fig. 3f, we detected that the number of CXCR4-positive cells in the M + UTMB 60 s group was significantly higher compared with the M group and the M + U 60 s group (*P* < 0.01).There was no significant difference in the number of CXCR4-positive cells between the M + UTMB 60 s group (13.31 ± 1.24%), the M + UTMB90s group (14.66 ± 1.35%) and the M + UTMB 180 s group (17.44 ± 1.16) (*P* > 0.05).

### Immunohistochemical (IHC) staining showed that CXCR4 was predominantly localized on the cell membrane and cytoplasm

**Fig. 4:**
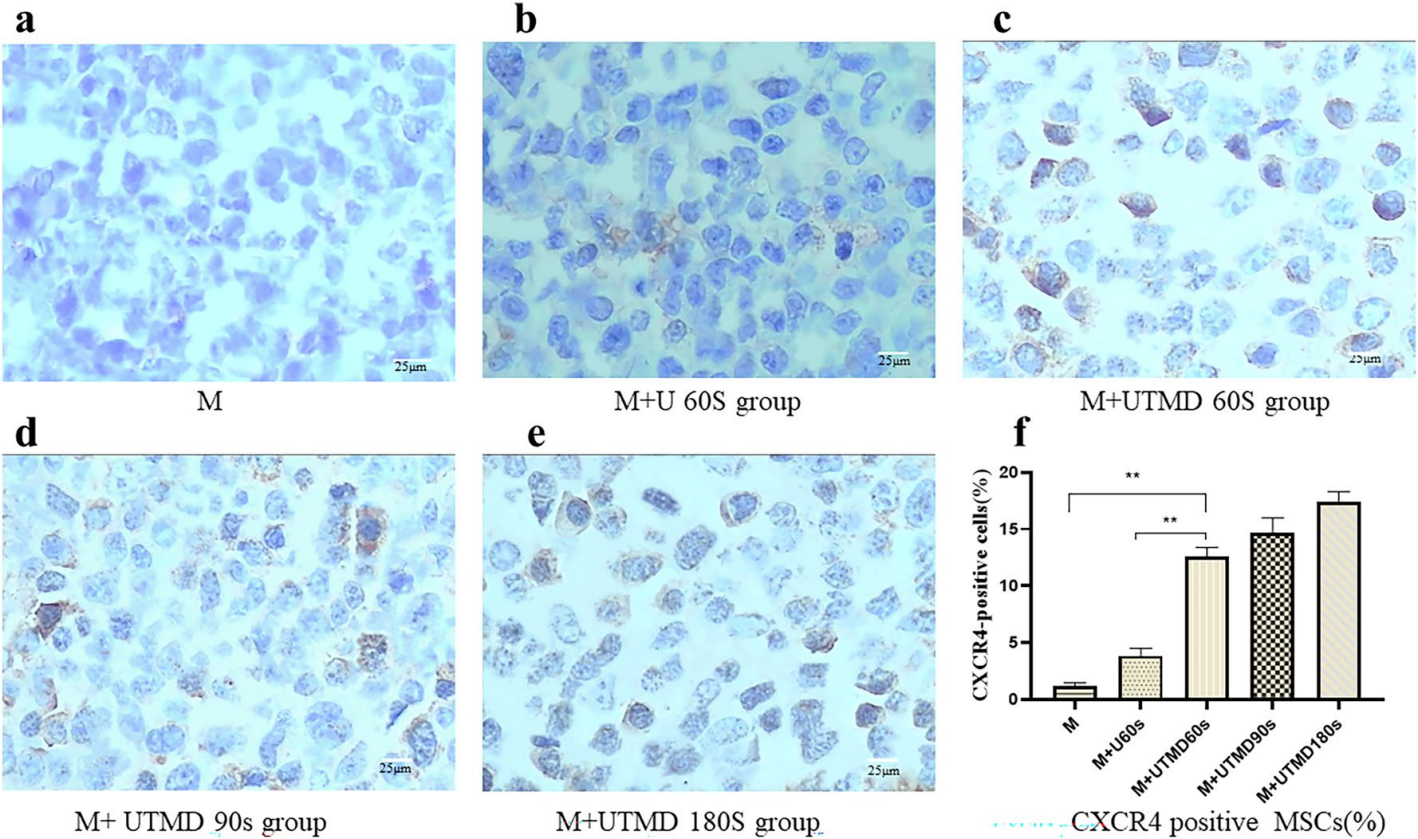
Expression of CXCR4 by IHC (n = 10). The surface of CXCR4 immunohistochemistry positive cells is brown (× 200, scale bar = 25 μm). **a**–**e** IHC of CXCR4 in the M group, M + U60s group, M + UTMD 60 s group, M + UTMD 90 s group, M + UTMD 180 s group. The number of CXCR4-positive cells was relatively smaller in the M group (**a**) and the M + U60s group (**b**), P > 0.05. The percentage of cells expressing surface CXCR4 in the M + UTMB60s group (**c**) higher than the M + U60s group (**b**) and the M group (**a**), P < 0.05. There was no significant difference in the number of CXCR4-positive cells between the M + UTMB60s group (**c**), the M + UTMB90s group (**d**) and the M + UTMB180s group (**e**), P > 0.05. **f** Quantification of CXCR4 expression was performed by Image-Pro Plus 6.0 software. All values are expressed as the mean ± SD. **P < 0.01; *n* = 10

The number of CXCR4-positive cells was relatively smaller in the M group and the M + U 60 s group. the percentage of cells expressing surface CXCR4 in the M + UTMB 180 s group (17.44 ± 0.85%) higher than the M + U 60 s group (3.86 ± 0.63%) and the M group (1.19 ± 0.31%) (Fig. 4a–e). As showed in Fig. 4f, we detected that the number of CXCR4-positive cells in the M + UTMB 60 s group was significantly higher compared with the M group and the M + U 60 s group (*P* < 0.01). There was no significant difference in the number of CXCR4-positive cells between the M + UTMB 60 s group (12.58 ± 0.81%), the M + UTMB 90 s group (14.72 ± 1.28%) and the M + UTMB180s group (17.44 ± 0.85%) (*P* > 0.05).

**Fig. 5:**
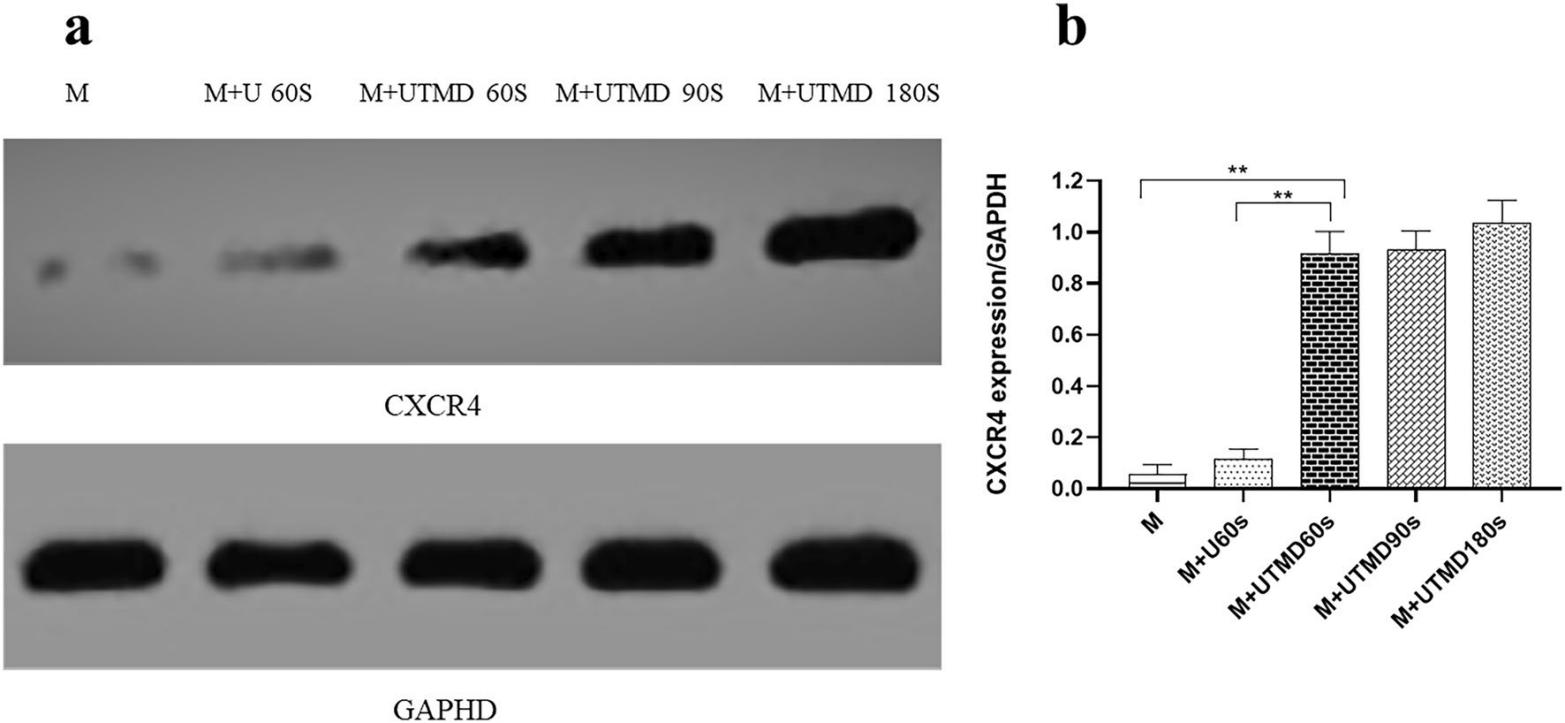
Expression of CXCR4 by WB (n = 6). **a** WB of CXCR4 in the M group, M + U60s group, M + UTMD 60 s group, M + UTMD 90 s group, M + UTMD 180 s group. **b** Quantification of CXCR4 expression was performed by Image J software. All values are expressed as the mean ± SD. **P < 0.01; *n* = 6

Western blot results showed that the level of CXCR4 was higher in the M + UTMB60s group (0.92 ± 0.09) compared to the M group (0.06 ± 0.04) and the M + U60s group (0.12 ± 0.04) (*P* < 0.01) and that the M + UTMB180s group (1.04 ± 0.09) had the highest levels compared to all other groups (Fig. 5a, b).

#### Effect of UTMB on cell viability

**Fig. 6:**
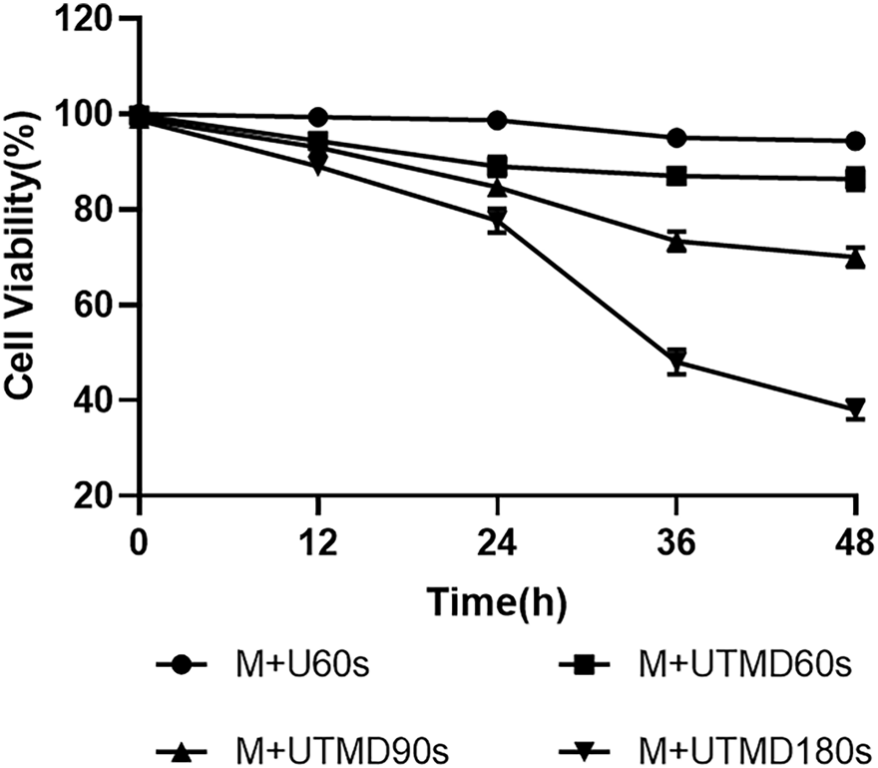
Trypan blue staining for cell viability. The results showed that ultrasound treatment for 60 s and UTMD treatment for 60 s had no significant effect on cell viability. With the further increase of UTMD treatment time from 60 to 180 s, cell viability decreases from 86.33 ± 1.89% to 38.00 ± 1.63% after 48 h of treatment. All values are expressed as the mean ± SD. **P < 0.01; *n* = 3

Trypan blue staining results showed that ultrasound treatment for 60 s and UTMB treatment for 60 s had no significant effect on cell viability. With the further increase of UTMB treatment time from 60 to 180 s, cell viability decreases from 86.33 ± 1.89 to 38.00 ± 1.63% after 48 h of treatment (Fig. 6).

### Assessment of transplanted MSCs homing to fibrotic liver

#### GFP labeled MSCs homing

**Fig. 7:**
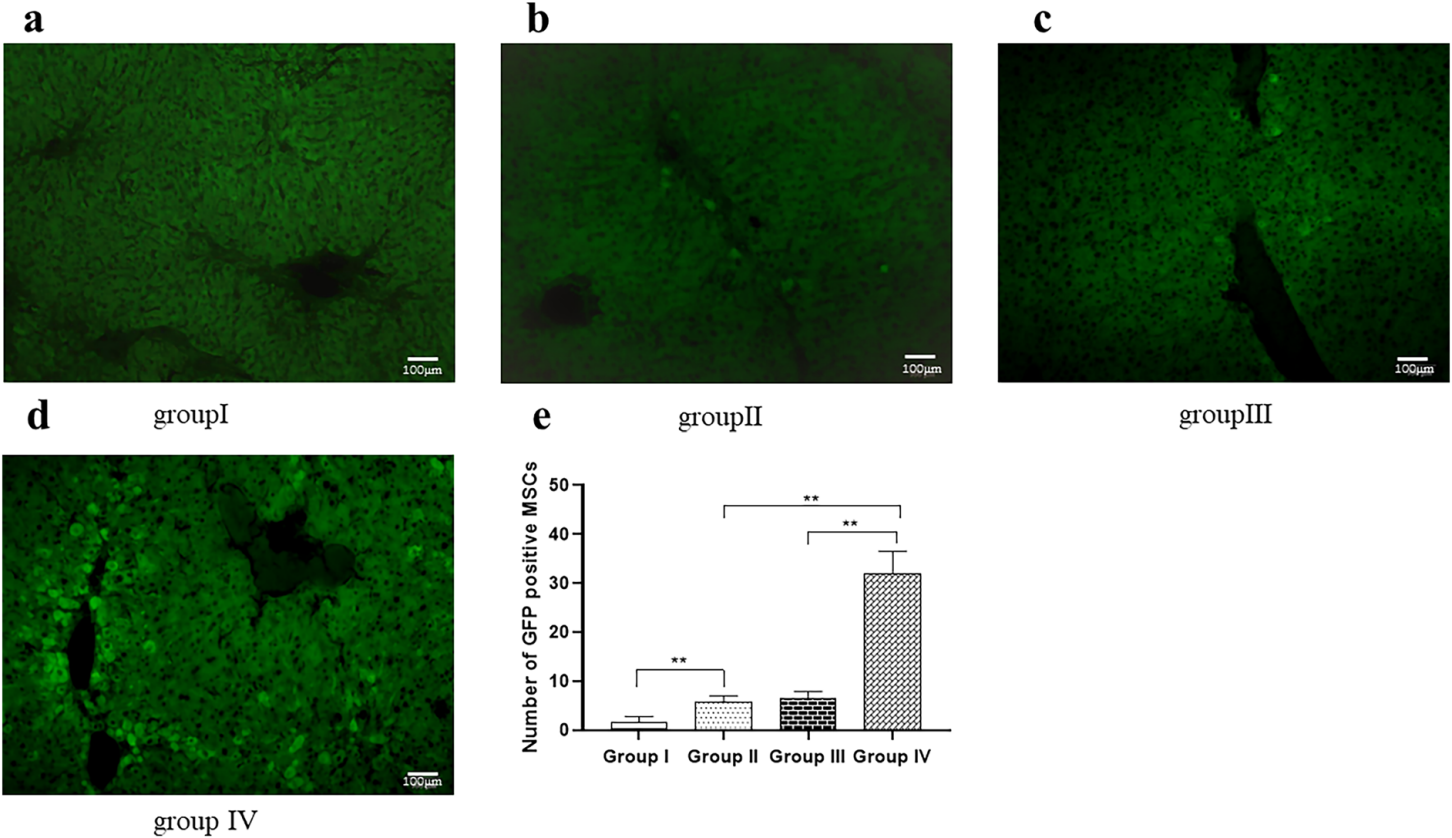
Representative photograph of GFP staining of MSCs in each group (n = 10). GFP-labeled MSCs were concentrated in the portal vein region under fluorescence microscopy images (×100, scale bar = 100 μm). **a** There are almost no GFP positive cells in the liver tissue and portal area of the group I. **b** There are a small number of GFP positive cells in the portal area of the group II. **c** There are GFP positive cells in the liver tissue and portal area of the group III. **d** There are a large number of GFP positive cells in the liver tissue and portal area of the group IV. **e** Quantitative analysis revealed that transplanted MSC in the group II (5.7 ± 1.26) was significantly increased compared to the group I (1.7 ± 1.09) (P < 0.01) and transplanted MSC in the group IV (31.9 ± 4.58) was significantly increased compared with other groups (P < 0.01). All values are expressed as the mean ± SD. **P < 0.01; *n* = 10

GFP-labeled MSCs were concentrated in the portal vein region under fluorescence microscopy images (Fig. 7a–d). We counted GFP-labeled cells in three randomly selected high-power fields (100×). The results showed that the number of GFP-positive cells in the group IV (31.9 ± 4.58) was significantly increased compared with the group II (5.7 ± 1.26) and the group III (6.4 ± 1.45). In addition, the number of GFP-positive cells in the group II (5.7 ± 1.26) was significantly increased compared with the group I (1.7 ± 1.09) (Fig. 7e).

#### IHC labeled MSCs homing

**Fig. 8:**
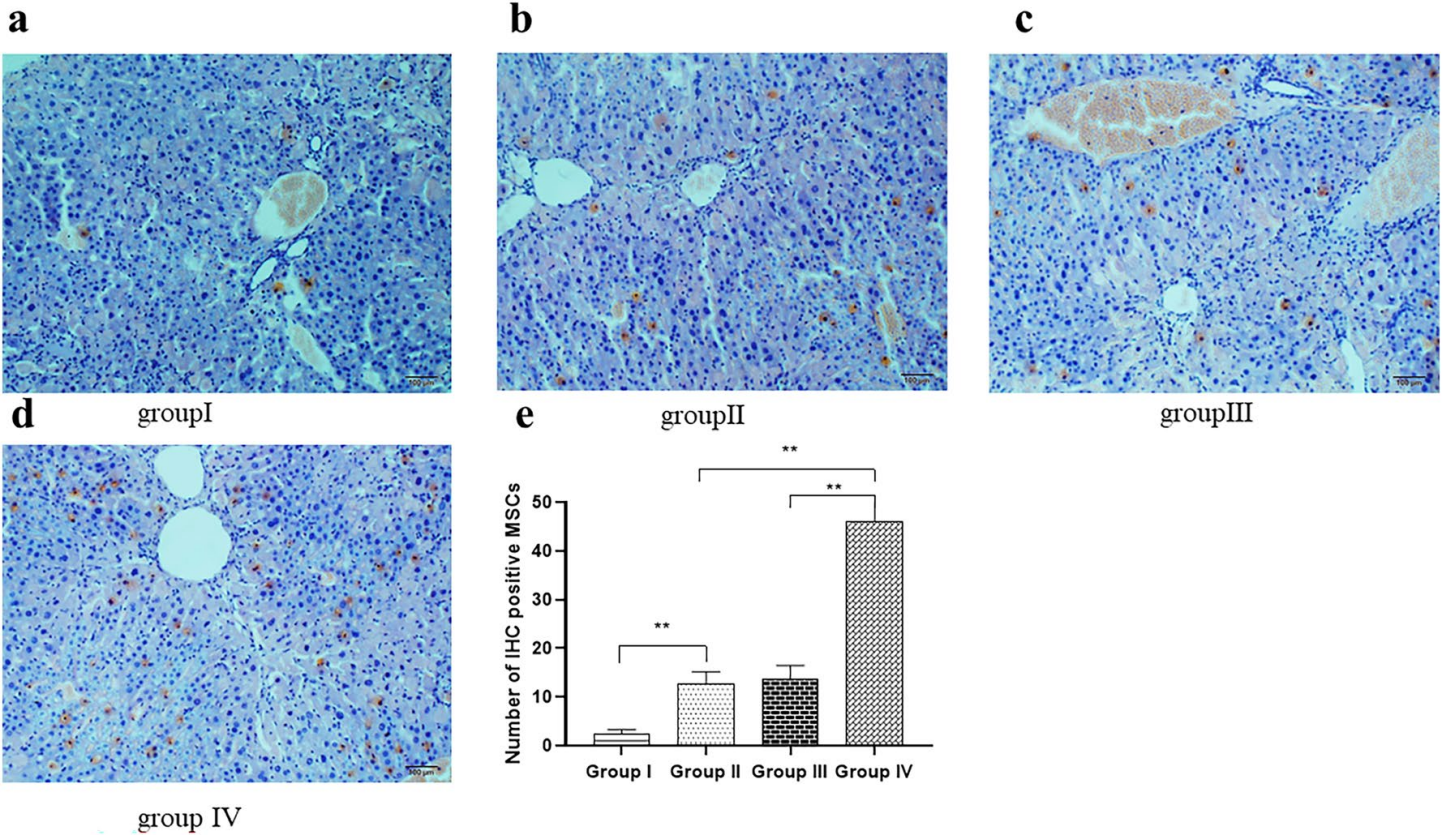
Representative photograph of IHC staining of MSCs in each group (n = 10). Anti GFP antibody (ab183734) -labeled MSCs were concentrated in the portal vein region under microscopy images (× 100, scale bar = 100 μm). **a** a few of IHC positive cells (brown) in the liver tissue and portal area of the group I. **b** There are a small number of IHC positive cells in the portal area of the group II. **c** There are IHC positive cells in the liver tissue and portal area of the group III. **d** There are a large number of IHC positive cells in the liver tissue and portal area of the group IV. **e** Quantitative analysis revealed that transplanted MSC in the group II (9.8 ± 2.53) was significantly increased compared to the group I (2.4 ± 0.84) (*P* < 0.01) and transplanted MSC in the group IV (37.1 ± 5.11) was significantly increased compared with other groups (*P* < 0.01). All values are expressed as the mean ± SD. **P < 0.01; *n* = 10

Anti GFP antibody (ab183734) -labeled MSCs were concentrated in the portal vein region under microscopy images (Fig. 8a–d). We counted IHC-labeled cells (brown) in three randomly selected high-power fields (100 ×). The results showed that the number of IHC-positive cells in the group IV (37.1 ± 5.11) was significantly increased compared with the group II (9.8 ± 2.53) and the group III (10.4 ± 2.37). In addition, the number of GFP-positive cells in the group II (9.8 ± 2.53) was significantly increased compared with the group I (2.4 ± 0.84) (Fig. 7e).

#### SDF-1 expression changes in liver tissues before and after UTMD stimulation

**Fig. 9:**
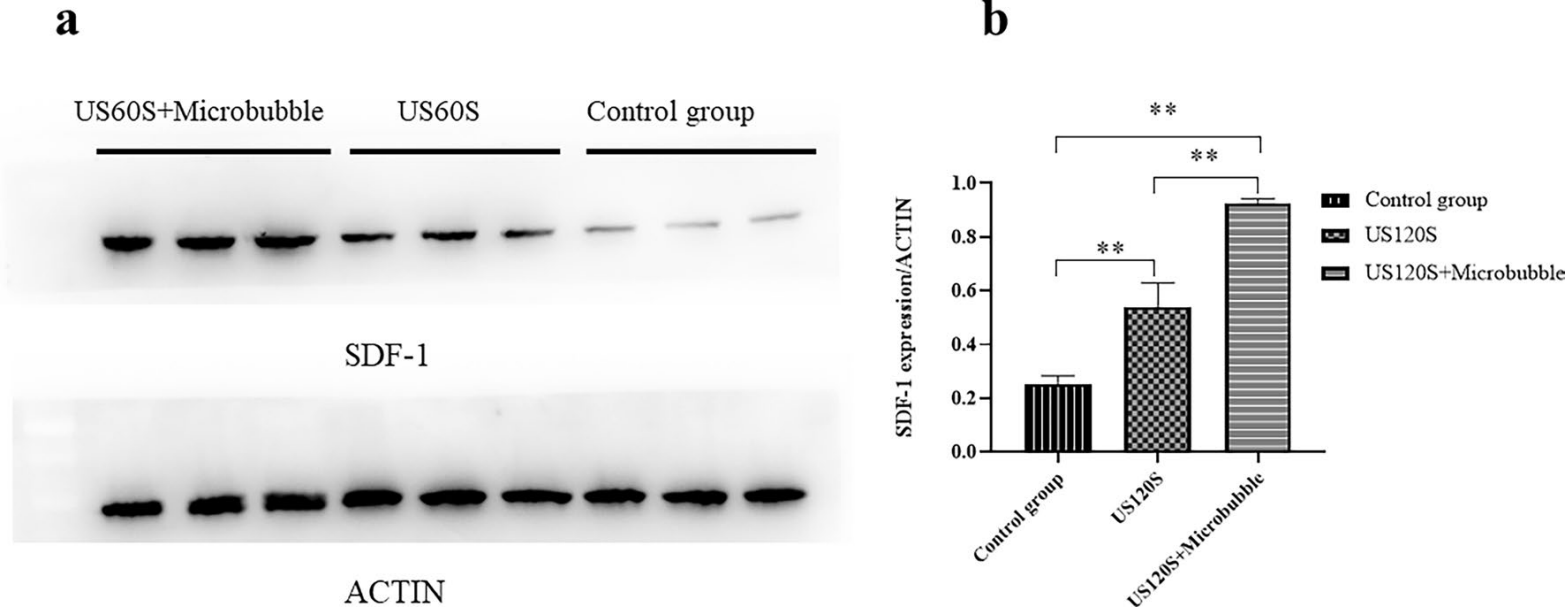
Expression of SDF-1 by WB (n = 3). **a** WB of SDF-1 in the Control group, US 60 s group, US + microbubble 60 s group, **b** Quantification of SDF-1 expression was performed by Image J software. All values are expressed as the mean ± SD. **P < 0.01; *n* = 3

SDF-1 is a natural ligand for CXCR4, and the concentration SDF-1 in damaged tissues can induce CXCR4-positive cell-specific migration. In the supplementary trial, we observed the effect of ultrasound microbubbles on SDF-1 expression in liver tissue, divided liver fibrosis model mice into a control group, ultrasound-stimulated group and the ultrasound plus microbubble group, ultrasound-stimulated group used Ultrasound probe was used at a frequency of 1 MHz and at strength of 1.0 W/cm^2^, and placed vertically above the liver of mice and Irradiated for 1 min, the ultrasound plus microbubble group was stimulated with tail vein injection of microvesicles microbubbles (SonoVue® sine, China, 1 ml) and the same intensity Ultrasound, 3 in each group. The results were shown in Fig. 9a, the liver tissue of the control group had a small amount of SDF-1 expression, and simple ultrasound stimulation could promote SDF-1 expression, and ultrasound combine with microbubbles could significantly increase the expression of SDF-1 (P < 0.01).

## Discussion

MSCs are a promising method for treating cirrhosis. However, present studies showed that MSCs transplanted into the body gathered in the lungs firstly, gradually increased in liver and spleen, and the liver has only few cells in liver after peripheral vein infusion of MSCs. Therefore, there is an urgent need to improve the homing ability and retention of systemically administered MSCs for the success of stem cell therapy in cirrhosis treatment.

Mesenchymal stem cell homing is a process in which autologous or exogenous mesenchymal stem cells migrate from vascular endothelial cells to targeted tissues and colonize survival under the influence of various factors, and inflammatory chemotaxis is the most important feature of mesenchymal stem cell homing [16]. Recent studies showed that SDF-1/ CXCR4 is the most important signaling pathway that mediates the specific migration of MSCs to damaged tissues. The previous studies showed that the long-term culture of MSCs may cause a marked decrease in CXCR4 expression and revoke MSCs’s chemotactic responsiveness to chemokines [17], which has limited the effective implementation of MSCs-based strategies.

In this study, we tested the surface CXCR4 expression on the MSCs treated by microbubble-mediated ultrasound irradiation and counted the number of homing MSCs labeled by green fluorescent protein in the fibrosis liver of mice. Flow cytometry analysis revealed increased expression of CXCR4 in the M + UTMB 60 s group, M + UTMB90s group, and M + UTMB 180 s group, as compared with the M group and M + U 60 s group (*P* < 0.01). There was no significant difference in the expression rate of CXCR4 between the M group and the M + U 60 s group (P > 0.05), which indicated that the unique physical effect of ultrasound could not increase the expression rate of CXCR4. In addition, different intensities of UTMB treatment increased the number of cells expressing surface CXCR4 in MSCs, Subsequent immunohistochemical and Western blotting results clarified the same conclusion. This indicates that UTMB upregulated the percentage of MSCs expressing surface CXCR4, which may benefit from the biological effects of ultrasound. There are two potential mechanisms. First, the sonoporation effect of UTMB can result in the construction of invertible tiny holes on the cell membrane, which enhances cell membrane permeability [18] and provide a physical deliver lane for the transmembrane protein CXCR4. Also, UTMB can promote the influx of calcium in BMSCs and increase mRNA transcription and protein expression of CXCR4. The latter may partly be caused by influx of calcium. Another important finding is that there was no significant difference in the expression rate of CXCR4 in the M + UTM B60s group, M + UTMB 90 s group, and M + UTMB 180 s group. The results showed that increasing the ultrasonic irradiation time did not significantly increase the expression of CXCR4 (*P* > 0.05). In this study, we refer to the irradiation conditions described in the literature [19], and found that ultrasound treatment for 60 s and UTMB treatment for 60 s had no significant effect on cell viability (*P* > 0.05). But, with the further increase of UTMB treatment time from 60 to 180 s, cell viability decreases from 86.33 ± 1.89 to 38.00 ± 1.63% after 48 h of treatment. The results indicate that excessive ultrasonic irradiation can cause irreversible damage to cells, in agreement with previously reported results. Therefore, appropriate UTMB treatment is very important for cell viability.

In recent years, with the wide application of ultrasound technology in the medical field and the development of ultrasound contrast agents, ultrasound-targeted microbubble destruction (UTMB) technology is a non-invasive treatment with high efficiency, safety, simple operation, and certain targeting. When microbubbles are exposed to the alternating compressional and rare fractional phases of ultrasound, they undergo volumetric expansion, coalesce and dissolution, a process called cavitation effect [20]. Som studies have observed that the microbubble volume expansion in the ultrasound field promotes the axial displacement of the blood vessels, leading to an increase in the vascular endothelial cell gap and promoting the passage of microvesicles through the endothelium into the tissue. Therefore, MSCs may enter the interstitial space due to increased vascular permeability under the effect of cavitation, but this remains to be confirmed by further research. However, the effect of UTMB on target organs or tissues does not only increase the permeability of blood vessels. The previous study demonstrated that UTMB can improve the expressions of VEGF, SDF-1, VCAM-1, which may be beneficial to the homing of MSCs. Recent evidence indicates that UTMB significantly increased the expression of E-selectin, VCAM-1, SDF-1 and VEGF in the renal microenvironment, and these cytokines transiently increased and then returned to normal levels. This means that UTMB can transiently cause slight damage and inflammatory response in the target organ, especially to promote the upregulation of SDF-1 expression plays a very important role in promoting the homing of mesenchymal stem cells to target organs. In our study, the number of GFP-labeled MSCs transplanted by UTMB-mediated pretreatment (group II) was significantly more successful than without pretreatment (group I) (*P* < 0.01), this indicates that UTMB can promote the homing of MSCs into fibrotic liver tissue by increasing the expression of CXCR4. Also, the number of GFP-labeled MSCs was much larger in the group IV than those of other groups (*P* < 0.01), the application of UTMB technology to the liver surface can have a better effect. Groups II and IV have large differences in the number of MSCs homing (Fig. 8), and there are three possible mechanisms. First, UTMB can increase the expression of CXCR4 both in vitro and in vivo. Second, UTMB may cause an increase in vascular endothelial cell space and promote MSCs to enter the tissue through the endothelium. Third, UTMB not only increases the expression of SDF-1 in tissues but also enhances the secretion of SDF-1 by MSCs [21].

At present, the research of ultrasound microbubbles combined with mesenchymal stem cells is mainly limited to diseases such as myocardial infarction [22], acute kidney injury [23] and cerebral ischemia [24], but little research has been done on cirrhosis. Karlas T [25] studies have shown that no mobilization of Bone marrow hematopoietic stem cell (BM-HSC) was observed in cases of mild, moderate or severe chronic liver damage, and speculated that the hepatic microenvironment may play two different roles in stem cell recruitment, as a suppressive agent in chronic damage and an inducer in acute conditions. This is similar to our studies. We saw very few MSCs homing in group I, but more MSCs were homing to the fibrotic liver after UTMB treatment. It could be concluded from this study that UTMB may promote the homing of mesenchymal stem cells to fibrotic liver by increasing the expression of CXCR4 on the surface of MSCs, which has important significance for improving the effect of MSCs on liver fibrosis.

However, our experiments still have some shortcomings, including the lack of detection of SDF-1, VEGF, VCAM-1 and other related indicators, and many subjects need to be improvement in the future, including the effects of acoustic intensity on cell viability and UTMB on human liver function.

## Conclusions

The combination of microbubble-mediated ultrasound irradiation and mesenchymal stem cells has never previously been used to improve the migration of stem cells in the fibrotic liver. To the best of our knowledge, this is the first report to do so. We demonstrated that UTMB may promote the homing of mesenchymal stem cells to fibrotic liver by increasing the expression of CXCR4 on the surface of MSCs, and this study enriches the treatment of liver fibrosis and has broad prospects in stem cell therapy on liver fibrosis.

## Materials and methods

### Cell

Mice bone marrow mesenchymal stem cells purchased from Cyagen Biosciences company. The cells were cultured and expanded in vitro. All cells used for the experiments were cultured in MSCs growth medium and incubated at 37 °C in 95% humidified air and 5% CO_2._ Flow cytometry (Beckman, USA) was performed to identify MSCs surface markers (CD29, CD44, CD34, CD45). We observed the infected MSCs by fluorescent microscope (OLYMPUS, IX71), and then analyzed the GFP labeling efficiency by flow cytometry (Beckman, Cyto Flex S).

The MSCs (1 × 10^6^ cells) at the third passage were suspended in 5 ml PBS and seeded into 25 cm^2^ culture flasks. Then, 5 μl microbubbles (SonoVue® sine, 2 × 10^11^/l) were slowly added to cell culture flasks. Each sample was gently mixed by rocking the plate before ultrasound irradiation. Diagnostic ultrasound device (Samsung Medison, H60, Korea) was used at a frequency of 1 MHz and the strength of 0.6 W/cm^2^, and irradiated for 60 s. The ultrasound probe was placed at the base of the water chamber, which was approximately 8 cm below the 25 cm^2^ culture flasks. To determine the effect of UTMB on MSCs membrane CXCR4 expression in vitro, we divided the experimental cells into five groups: control group (MSCs group, M), ultrasonic irradiation 60 s group (M + U60s), ultrasonic irradiation 60 s combined with microbubble group (M + UTMB60s), ultrasonic irradiation 90 s combined with microbubble group (M + UTMB90s) and Ultrasonic irradiation 180 s combined with Microbubble group (M + UTMB180s). After ultrasound irradiation, the culture flasks were placed in an incubator at 37 °C in 95% humidified air and 5% CO_2_. Flow cytometry (FCM), Western-blot (WB) and Immunohistochemistry (IHC) analysis were conducted to assess CXCR4 expression efficiency at 24 h post-treatment.

### Animal models

Mice (BALB/c, aged 7–9 weeks) were purchased from the Animal Experimental Center of Anhui Medical University. All experimental procedures were following the Animal Care Unit and Use Committee of Anhui Medical University, and the experimental protocol was approved by the ethics committee of the 901th hospital of PLA. Nine-week-old male BALB/c mice were treated with an Intraperitoneal injection of 1.6 ml/kg CCl_4_ (Qiang Sheng, China) dissolved in olive oil (1:4) twice a week for 7 weeks, and always use 30% ethanol solution and food made available ad libitum. In the training set, stage of fibrosis for each liver biopsy specimen were determined by one expert liver pathologists using metavir systems. The metavir fibrosis stage of the portal tract was as follows: 0, no fibrosis; 1, enlarged portal tract without septa; 2, enlarged portal tract with rare septa; 3, numerous septa without cirrhosis; 4, cirrhosis.

40 BALB/c mice were successfully modeled and randomly divided into four groups: the normally cultured MSCs [1 × 10^6^ cells suspended in 1 ml Phosphate balanced saline (PBS)] transplanted by the tail vein injection was set as control group (group I, n = 10), the MSCs treated with UTMB for 60 s group (group II, n = 10), MSC treated with ultrasound microbubbles for 60 s and combined with in vivo ultrasound radiation therapy group (group III, n = 10), while MSC treated with ultrasound microbubbles for 60 s, combined with in vivo ultrasound radiation and microbubble therapy group (group IV, n = 10). In group III and IV, immediately after MSCs or/and microbubbles (SonoVue®, 1 ml) were injected through the tail vein, diagnostic ultrasound (Samsung Medison,H60, Korea) was used at a frequency of 1 MHz and at strength of 1.0 W/cm^2^, and placed vertically above the liver of mice and irradiated for 2 min. The mice of group III were treated with the same sonication as group IV.

### Flow cytometry analyses

Expression of cell surface CXCR4 on MSCs was detected with PE-conjugated monoclonal anti-mice CXCR4 antibody (Biolegend, China). Briefly, cells were resuspended in 1 × Binding Buffer at a concentration of 10^6^ cells/ml. Five microliters of PE-conjugated monoclonal anti-mice CXCR4 antibody were added and incubated with cells at room temperature for 15 min. Labeled cells were then analyzed by a Cytoflex S flow cytometer with the use of Cell Quest software (Beckman Coulter, USA).

### Immunohistochemistry analyses

Cells were resuspended with 100% ethanol and then centrifuged for 5 min at 2500 rpm at 4 °C. Fixed in 10% formaldehyde for 2–3 h, embedded in paraffin, and then sectioned into 4 µm slices. Then they were incubated with 1:100 rabbit polyclonal antibody against CXCR4 (4 A Biotech, China) for 1 h. After that, they were washed in PBS and incubated with horseradish peroxidase-labeled goat anti-rabbit IgG (Absin, China) for 30 min. Control experiments included the omission of either the primary or secondary antibody. After counterstaining with hematoxylin, the slices were observed under a light microscope. Ten random fields of each section were photographed and a semiquantitative evaluation of CXCR4 expression was performed. Integrated optical density (IOD) of CXCR4 expression was analyzed using Image-Pro Plus 6.0 software (Media Cybernetics, USA).

### Western blotting analyses

Cell membrane proteins were isolated using a membrane protein extraction kit according to the manufacturer’s instructions (Beyotime, China). Briefly, cells were incubated with lysis buffer at 4 °C for 30 min and then centrifuged for 30 min at 12,000 rpm at 4 °C. Protein concentration was determined by bicinchoninic acid (BCA) protein assay kit. Denatured proteins (20 mg) were separated by SDS-PAGE and transferred onto a PVDF membrane. The membrane was blocked with skim milk dissolved in TBST at room temperature for 2 h and then incubated overnight at room temperature with rabbit anti-CXCR4 primary antibody at a 1:500 dilution (Abcam, UK) and finally incubated with goat anti-rabbit HRP-conjugated secondary antibody at a 1:1000 dilution (Beyotime, China) for 1 h the next day. CXCR4 was normalized to GAPDH (Jian Cheng, China). We quantitatively analyze the signal of protein band by chemiluminescence image processing system (tanon-5200 multi, China). The relative levels of CXCR4 are denoted by the ratio of CXCR4/GAPDH. SDF-1 was normalized to ACTIN (Jian Cheng, China). The relative levels of SDF-1 are denoted by the ratio of CXCR4/ACTIN.

### Trypan blue cell viability assay

Cells in the different treatment groups were digested with 0.25% trypsin at 0, 12, 36, and 48 h, and total cell counts were determined using a hemocytometer chamber after staining the cells with 0.4% trypan blue (Ba So, China). Three minutes later, living (not stained) and dead cells (dyed blue) were counted. Live cell rate (%) = the number of living cells/total cell number.

### Detection of implanted MSCs

All mice were sacrificed 24 h after the cell transplantation, the survival of implanted cells was determined by the number of GFP-positive cells in section (4 μm) made from fibrotic liver under a fluorescent microscope.

### Statistical analysis

All data are expressed as mean ± SD. The SPSS 23.0 software was used for statistical analyses. When the data variance is homogeneous, comparisons among multiple groups were tested using one-way analysis of variance (ANOVA) and follow-up comparisons between two groups were conducted using the Bonferroni method. When the data does not conform to the normal distribution or the variance is not uniform, comparisons among multiple groups were tested using Kruskal–Wallis H Test and follow-up comparisons between two groups were conducted using the Bonferroni method. Statistical *P* values < 0.05 were considered significant.

## Data Availability

The original contributions presented in the study are included in the article, further inquiries can be directed to the corresponding author/s.
